# Special Issue: “Innate Immunity to Virus Infection, 1st Edition”

**DOI:** 10.3390/v15102060

**Published:** 2023-10-07

**Authors:** Congcong Wang, Feng Ma, Caijun Sun

**Affiliations:** 1 School of Public Health (Shenzhen), Shenzhen Campus of Sun Yat-sen University, Shenzhen 518107, China; wangcc5@mail2.sysu.edu.cn; 2 Key Laboratory of Tropical Disease Control, Sun Yat-sen University, Ministry of Education, Guangzhou 510080, China; 3 National Key Laboratory of Immunity and Inflammation, and CAMS Key Laboratory of Synthetic Biology Regulatory Elements, Suzhou Institute of Systems Medicine, Chinese Academy of Medical Sciences & Peking Union Medical College, Suzhou 215123, China

Frequent outbreaks of emerging and re-emerging pathogenic viruses have become one of the major challenges for global public health. As the first line of defense, the innate immune system plays a vital role in fighting the invasion of pathogenic microorganisms. In response to viral entry into the host cell, pattern recognition receptors (PRRs) recognize the pathogen-associated molecular patterns (PAMPs) of viruses and then activate innate immune signaling pathways, which subsequently trigger the expression of numerous interferon-stimulated genes (ISGs) to exert direct antiviral effects [1,2]. Meanwhile, many viruses have developed various strategies to escape the innate immunity [3]. To deeply understand this complex interplay, we launched this Special Issue to gather novel knowledge about innate immunity and viral infections, and we hope that these latest studies can provide insights into developing antiviral therapeutics and vaccines.

Porcine epidemic diarrhea virus (PEDV) is a positive-sense single-stranded RNA virus that belongs to a coronavirus family. Many studies have shown that several PEDV proteins, including nsp1, nsp3, nsp5, nsp8, nsp14, nsp15, nsp16, E, M, and N, can restrict host IFN signaling. The research article by Zhang et al. investigated multiple PRR-mediated signaling pathways involved in the anti-PEDV responses. The innate immune signaling adaptors TRIF, MAVS, and STING exhibit blatant anti-PEDV activity, according to the authors’ screening of porcine innate immune signaling adaptors’ antiviral activity using transfected Vero cells. To further confirm it, knockdown or knockout of endogenous TRIF, MAVS, and STING promoted PEDV replication via siRNA and CRISPR approaches [4]. These results show that multiple porcine PRR-mediated signaling pathways are involved in PEDV recognition and defense, expanding our understanding of innate immunity responses to PEDV infection.

Recently, it has been important to study how the noncanonical NF-κB pathway participates in innate immunity. Bisom et al. conducted research to investigate the function of RIOK3 during Rift Valley Fever virus (RVFV) infection. They found that RVFV infection activated the noncanonical NF-κB pathway to weaken the antiviral IFN signaling response due to the production of the alternatively spliced RIOK3 X2 isoform, which encodes a truncated RIOK3 [5]. This finding will be helpful for deeply understanding the pathogenesis of RVFV through the regulation of the noncanonical NF-κB pathway to enhance viral replication.

Yao et al. reported their data on the crucial role of pulmonary microvascular endothelial cells (MVECs) in regulating inflammation during highly pathogenic porcine reproductive and respiratory syndrome virus (HP-PRRSV) infections. They reported that HP-PRRSV primarily induced virus-associated innate immune responses, whereas bacterial lipopolysaccharide (LPS) was responsible for the inflammatory response. HP-PRRSV infection exacerbated the inflammatory response due to secondary bacterial infections [6]. These results supported the importance of pulmonary MVECs in lung inflammation injury by primary HP-PRRSV infection and secondary bacterial infection.

Wen et al. focused on fusing the autophagosome-associated LC3b protein to the nucleocapsid (N) antigen, which is expected to improve the SARS-CoV-2-specific T cell functionality for developing the next-generation vaccine against SARS-CoV-2 variants. They concluded that the N-LC3b protein group can simultaneously secrete multiple cytokines (IFN-γ+/IL-2+/TNF-α+), improving T cell proliferation, especially for CD8+ T cell responses. In addition, their strategy was also induced a robust humoral immune response against the N antigen [7].

The role of IFITM3 in the SARS-CoV-2 pandemic is still controversial. Xu et al. reported their data on the association between IFITM3 and the risk of acquiring a SARS-CoV-2 infection. They demonstrated that IFITM3 inhibited SARS-CoV-2 infection by preventing virus entry, which is dependent on the first 21 amino acids of IFITM3. In addition, they also found that the rs12252 CC genotype of IFITM3 increased the risk of acquiring a SARS-CoV-2 infection and the decreased level of neutralizing antibodies against SARS-CoV-2 [8].

Another five review publications summarized the state-of-the-art research on the interplay between viruses and host innate immunity. Alves et al. summarized how placental cells engaged in innate immune responses play roles in response to Dengue virus (DENV) and chikungunya (CHIKV) infections [9]. Li et al. reported the roles of various well-known viruses in hijacking cytoskeletal structures and the accompanying antiviral responses [10]. Roldan et al. described the comprehensive understanding of the possible mechanisms of anti-cytokine autoantibody production, which could improve the approach to treating some infections, not only targeting pathogens but as a treatment for some autoimmunity patients [11]. Benzarti et al. discussed the complicated roles and expression patterns of interleukins, chemokines, and tumor necrosis factor superfamily ligands associated with West Nile virus (WNV) infection and pathogenesis [12]. Min et al. discussed the regulatory role of IFN-induced noncoding RNA (ncRNA) in antiviral innate immunity, aiming to improve our understanding of ncRNAs and provide insights for the basic research of antiviral innate immunity [13].

In conclusion, these ten articles published in this Special Issue should improve our understanding on the complex interactions between viral infections and host innate immune responses. These findings provide a summary of the most updated findings on PEDV, RVFV, PRRSV, SARS-CoV-2, DENV, CHIKV, and WNV, which are crucial for the subsequent development of novel approaches to prevent and control viral infections.
